# Comparative Analysis of the Most Important Cardiovascular Risk Factors Based on Cross-Sectional Studies in the Population of Latvia

**DOI:** 10.3390/medicina58050643

**Published:** 2022-05-06

**Authors:** Andrejs Erglis, Iveta Bajare, Sanda Jegere, Iveta Mintale, Juris Barzdins, Artis Luguzis, Peteris Apinis, Anda Caksa, Iveta Gavare, Vilnis Dzerve

**Affiliations:** 1 Institute of Cardiology and Regenerative Medicine, University of Latvia, LV-1004 Riga, Latvia; iveta.bajare@kardiologija.lv (I.B.); sanda.jegere@stradini.lv (S.J.); iveta.mintale@stradini.lv (I.M.); peteris.apinis1@gmail.com (P.A.); anda.caksa@gmail.com (A.C.); vilnisdzerve@inbox.lv (V.D.); 2 Latvian Centre of Cardiology, Pauls Stradins Clinical University Hospital, LV-1002 Riga, Latvia; 3 Faculty of Physics, Mathematics and Optometry, University of Latvia, LV-1004 Riga, Latvia; juris.barzdins@lu.lv (J.B.); artis.luguzis@lu.lv (A.L.); 4 Latvian Medical Association, LV-1010 Riga, Latvia; 5 Centre for Disease Prevention and Control, LV-1005 Riga, Latvia; iveta.gavare@spkc.gov.lv

**Keywords:** cardiovascular risk factors, cross-sectional epidemiological survey, epidemiology

## Abstract

*Background and Objectives*: The aim of the study was to analyze the prevalence of cardiovascular risk factors (RFs) in Latvia from the population-based cross-sectional study performed in 2019–2020 and to compare the results with a similar study done in 2009–2010. *Materials and Methods*: The target sample of 6000 individuals representing a cross-section of Latvia’s inhabitants (aged 25–74) was formed using stratified two-stage cluster sampling. The survey had two components: (1) an interview using a pre-specified questionnaire and (2) physical examination (height, weight, arterial pressure) and collection of venous blood samples to measure levels of fasting glucose (Glu), total cholesterol (TC), high and low-density lipoprotein cholesterol (HDL-C/LDL-C), and triglycerides (Tg). In total, 4070 individuals were interviewed (32% non-response), from which 2218 (55%) individuals underwent physical examination and collection of blood samples. *Results*: The most frequently observed RFs were high LDL-C (62.0%), smoking (45.3%), and arterial hypertension (36.8%), while the prevalence of self-reported high cholesterol and hypertension was 19.3 and 18.6%, respectively. A decrease in the prevalence of hypertension, high LDL-C, and Glu was noted. Smoking decreased in younger men. The mean number of five most important cardiovascular RFs was 2.0 (95% confidence interval (CI) 2.0, 2.1); 2.3 (95% CI 2.2, 2.4) for men and 1.8 (95% CI 1.7, 19) for women. The average number of RFs has decreased by 0.3 in 10 years, t(5883) = −7.2, *p* < 0.001. *Conclusions*: Although the prevalence of cardiovascular RFs remains noteworthy, an improvement in the risk profile of the Latvian population has been observed over the past decade. The study shows subjective self-underestimation of cardiovascular risk.

## 1. Introduction

An increasing prevalence of non-communicable diseases is a major public health concern in many countries and in Latvia. The health profile of the Latvian population reflects high morbidity and mortality due to cardiovascular diseases. During the last ten years, according to Latvian mortality statistics, cardiovascular diseases (CVD) accounted for 52–57% of all deaths, and the mortality rate remained to be high: 750.7 and 783.9 per 100,000 from 2009 to 2019, respectively [1]. Public health is a continuously changing system that can be understood by keeping track of its dynamics data. It is necessary to watch trends, react proactively to them, and detect possible future challenges to be prepared in advance. The data can be obtained in epidemiological studies that meet two main criteria: a broad cross-section of the population and proper methodology.

The previous population-based cross-sectional study of CVD RFs in Latvia that corresponded to internationally established standards and protocols took place in 2009–2010 [2]. The present study aimed to analyze the prevalence of the most important cardiovascular RFs identified in the nationwide epidemiological study in 2019–2020 according to the guidelines of the European Society of Cardiology [3,4,5]. The results were compared to the data from the above-mentioned study of 2009–2010 to assess the possible changes in the RFs prevalence.

## 2. Materials and Methods

### 2.1. Design

This cross-sectional population-based survey was conducted in 2019–2020. The population in interest was all adults aged 25–74 living in Latvia. To ensure representativeness, the population of 6000 people was formed as a result of computerized random selection (probability or simple random sampling) from the population and housing data base of the Central Statistical Bureau of Latvia (1,209,756 persons). A stratified systematic random sampling design was used. Strata were formed according two characteristics—sex (2 groups) and age (10 groups with an interval of 5 years: 25–29, 30–34, 35–39, etc.). There were 300 individuals in each of the strata. A systematic sample of persons was used in each strata, individuals were sorted by place of residence before the sample (hierarchical arrangement by region of the person’s place of residence, administrative territory, geographical coordinates). The Ethics Committee of the Institute of Cardiology and Regenerative Medicine, University of Latvia, approved the survey design and methodology (No. 2-260918, 26 September 2018). A Steering Committee (3 people) was set up to supervise the study.

### 2.2. Data Collection

The fieldwork of the survey was planned and organized by the Central Statistical Bureau in collaboration with the Institute of Cardiology and Regenerative Medicine, University of Latvia. Trained interviewers performed recruitment of the subjects. The subjects from a definite administrative area were informed about the visit time in advance by mail and/or telephone. The participants were visited, questioned, and asked to have a blood test at the nearest certified laboratory. The examination was free of charge and lasted for an average of 40–50 min.

The study consisted of two basic parts: a subjective investigation (questionnaire) and an objective examination. The questionnaire consisted of 16 sections, the questions divided into several categories: sociological (parts 1–2), those focused on well-known risk factors (parts 3–8), and those focused on specific diseases and syndromes (parts 9–16). The questions were formed according to a logical transition principle, depending on the provided answers. Data on the socio-economic status, the prevalence of smoking, alcohol consumption, diet, physical activity, health self-assessment, mental health, etc. were obtained using World Health Organization CINDI Health Monitor Survey [6] and HADS questionnaire (Hospital Anxiety and Depression Scale) [7] in face-to-face interviews. The questionnaire was pre-tested in January–February 2019 in a pilot study where 80 respondents were randomly involved. The results were summarized, analysed and appropriate corrections were made in the questionnaire.

The objective examination included: a double measurement of arterial blood pressure (BP), body weight, height, blood lipid profile, and blood glucose. The weight was measured by a digital scale and the height—by a stadiometer attached to a wall. The height and weight were used to calculate the body mass index (BMI) in kg/m^2^. The arterial BP was measured by an automated validated device (OMROM M6 Comfort) [8] with the subject in a sitting position, on the subject’s right arm, after having a rest for at least 5 min. The three measurements were taken at an interval of 2–3 min. The last two measurements were averaged for the analysis.

Venous blood samples were collected for the total cholesterol (TC), low-density lipoprotein cholesterol (LDL-C), high-density lipoprotein cholesterol (HDL-C), triglycerides (Tg), and fasting glucose (Glu) in the morning after an overnight fasting period. The analysis of these samples was done in certified laboratories of one laboratory chain [9]. Glucose was measured by enzymatic hexokinase/glucose-6-phosphate dehydrogenase (G6PD) method; a lipid panel (Chol, Tg, HDL, LDL) was tested using the enzymatic colorimetric method.

### 2.3. Risk Factors

The following most important cardiovascular RFs were analyzed:

- high BP; defined as systolic BP of 140 mmHg or more, and/or diastolic BP of 90 mmHg or more; the subjects were classified as having hypertension if their systolic BP was over 140 mmHg, and/or the diastolic BP over 90 mmHg, and/or if they were on pharmacological treatment for hypertension, including those whose hypertension was controlled (i.e., lower than 140/90 mm Hg);
- overweight and obesity; BMI cut-off points of 25 and 30 kg/m^2^ were used to determine the overweight and obese subjects, respectively;
- smoking; according to the smoking status, participants were classified into three main categories: daily smokers, ex-smokers, and never-smokers. Daily smokers were defined as smokers who continue smoking now; ex-smokers—smokers who have quitted smoking at least 6 months before examination;
- dyslipidaemias; the cut-off points of dyslipidaemias were as follows: TC ≥5.0 mmol/L, LDL-C ≥ 3.0 mmol/L, HDL-C ≤ 1.0 mmol/L for men, and ≤1.2 mmol/L for women, Tg ≥ 1.7 mmol/L, Glu ≥ 5.6 mmol/L.

The mean number of the cardiovascular RFs reported corresponds to the following five RFs: (1) systolic BP ≥ 140 mmHg, and/or diastolic BP ≥ 90 mmHg; (2) BMI ≥ 25 kg/m^2^; (3) smoking daily; (4) LDL-C ≥ 3.0 mmol/L; (5) Glu ≥ 5.6 mmol/L.

### 2.4. Statistical Analysis

The sample obtained was weighted using calibration weights to match the distribution of the target population by age, sex, and administrative area.

The prevalence estimates of cardiovascular RFs were presented as percentages with 95% confidence intervals (95% CI). Differences in the prevalence between sexes, age groups, and studies (2009–2010 vs. 2019–2020) were evaluated using the Chi-squared test. For each participant, the number of cardiovascular RFs was determined. The mean (95% CI) of the number of cardiovascular RFs was reported and compared between groups using a two-sample *t*-test. The association between the mean number of RFs and ten 5-year age groups was evaluated using the Spearman rank correlation (r). We used an alpha level of 0.05 for all statistical tests.

The analysis was performed in R Statistical software (version 4.0.5 [10]), Foundation for Statistical Computing, Vienna, Austria). Functions from the survey package [11] were used to account for calibration weights when estimating proportions, mean values, and confidence intervals from a weighted sample.

## 3. Results

Out of 6000 persons in the target sample, study interviews were received from *N* = 4070 (67.8% respondence) respondents; 23.9% were born in Riga, 64.4% in other regions of Latvia, and 11.7% outside Latvia. Twenty-one nationalities were represented: 63.7% of the respondents were Latvian, 25.7%—Russian, and 10.6%—other.

The distribution of the respondents by age and sex is shown in Table 1 and by the socio-demographic characteristics in Table 2. The most common self-reported risk factors were smoking (30.0%), high cholesterol (19.3%), and hypertension (18.6%). Smoking prevalence was higher among men than women. In contrast, women had a statistically significant higher prevalence of hypertension, hypercholesterolemia, diabetes, and physical inactivity (Table 3).

Physical examination, including body weight, blood pressure and heart rate measurements and the blood test, were obtained from 2218 (37.0% of the target sample) respondents, i.e., 54.5% of the interviewed respondents. Results regarding the prevalence of objectively measured RFs in the general population and in various age-sex groups are shown in Table 4 and Table 5. The most common observed RFs were high LDL-C (62.0%), smoking (45.3%), and arterial hypertension (36.8%). In contrast to self-reported risk factors, hypertension was more prevalent in men when compared to women (40.3% vs. 33.8%, *p* = 0.003). The analysis of the prevalence of main risk factors were analyzed also in all respondents vs. respondents with laboratory tests and there was no significant difference in the characteristics of the population.

### 3.1. Cardiovascular Disease Risk Factors

**Figure 1:**
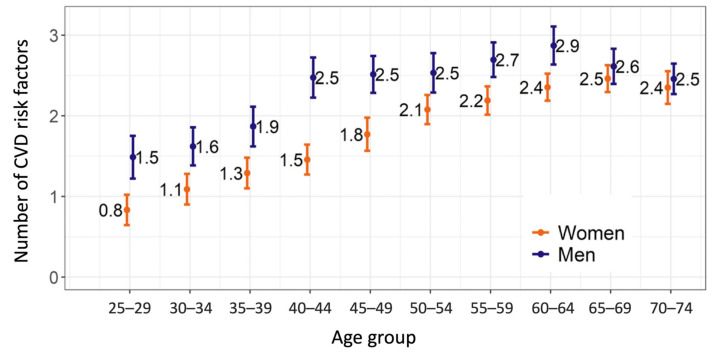
Mean number (95% CI) of CVD risk factors by gender/age.

The mean number of the most important cardiovascular RFs was 2.0 (95% CI 2.0, 2.1) per person for the overall study population. The male group had a significantly greater mean number of RFs (2.3 (95% CI 2.2, 2.4)) than the female group (1.8 (95% CI 1.7, 1.9), t(2187) = 8.8, *p* < 0.001). The number of RFs increased with age both in men and women; a positive correlation between the 5-year age group and the mean number of RFs was found in men (r = 0.80, *p* = 0.005) and in women (r = 0.98, *p* < 0.001). In the age group of 65 years and more, the mean number of RFs decreased in men and stabilized in women (Figure 1). In our study 6.1% of men and 14.3% of women did not have any of the traditional risk factors.

### 3.2. High Blood Pressure and Hypertension

The mean systolic blood pressure was 124.4 ± 19.4 mm Hg (129.3 ± 18.2 in men vs. 120.3 ± 19.4 mmHg in women, *p* < 0.001). The mean diastolic blood pressure was 81.2 ± 12.7 mm Hg (82.5 ± 12.8 in men vs. 80.0 ± 12.6 mmHg in women, *p* < 0.001).The prevalence of increased blood pressure (systolic ≥ 140 mmHg, and/or diastolic ≥ 90 mmHg) was in 28.0% of the general study population, 33.3% of male population, but only 23.5% of female population (*p* < 0.001).

The proportion of individuals with increased blood pressure increased considerably with age in both sex (Table 5). Furthermore, there was a positive correlation between the age group and the prevalence of increased blood pressure both in men (r = 0.99, *p* < 0.001) and women (r = 0.96, *p* < 0.001).

Additionally, the prevalence of hypertension was analyzed. According to study definition, 45.5% respondents had hypertension while the prevalence of self-reported hypertension was 18.6%.

### 3.3. Body Mass Index

The mean BMI was 27.5 ± 5.6 kg/m^2^ (27.8 ± 5.3 in men vs. 27.2 ± 5.9 kg/m^2^ in women, *p* < 0.001). The prevalence of overweightness (BMI 25–29.9 kg/m^2^) and obesity (BMI ≥ 30 kg/m^2^) among the respondents was 34.3 and 29.1%, respectively. Notable sex differences were revealed regarding overweight: 40.6 in men and 28.8% in women (*p* < 0.001). Overweight and obesity increased with age among women, while overweight did not change with age among men (Table 5).

### 3.4. Smoking

31.5% of men and 15.2% of women reported being daily smokers. The prevalence of daily smoking decreased with age among women, while among men it was stably high at the age of 25–64 and decreased afterwards (Table 5). The overall prevalence of smoking for at least 1 year during a lifetime was 45.3%; however, a noticeable difference in the proportion between men (60.8%) and women (31.7%) was observed (*p* < 0.001). Additional survey questions showed that the respondents who were smokers smoked 13 cigarettes with a filter per day on average. A proportion of 18.6% of the respondents who had been smokers for at least 1 year had initiated smoking when less than 16 years old; 63.6% had started smoking aged 16 to 20; 12.5%—aged 21 to 25; 5.4%—older than 26. A proportion of 52.8% of smokers would like to stop smoking; 27.5% of the respondents were exposed to passive smoking at work, home, or elsewhere.

### 3.5. Lipids

The mean TC was 5.4 ± 1.2 mmol/L (5.3 ± 1.1 in men vs. 5.6 ± 1.2 mmol/L in women, *p* < 0.001), but LDL-C was 3.4 ± 1.1 mmol/L (3.4 ± 1.0 in men vs. 3.4 ± 1.1 mmol/L in women, *p* > 0.9). The prevalence of increased TC level was comparatively high both in men (60.7%) and women (65.4%) (*p* = 0.036). The prevalence of persons with LDL-C ≥ 3.0 mmol/L was also high but slightly more similar in both sex groups: 63.1% in men and 61.1% in women (*p* = 0.379). The prevalence of individuals with LDL-C below 2.0 mmol/L was 7.9% in the overall study population; no significant difference between sex groups was observed (*p* = 0.870). The prevalence of decreased HDL-C was 14.0% in the general study population; a higher proportion was observed in men (16.9%) than women (11.5%; *p* = 0.001). Around one fourth (24.7%) of the study population had hypertriglyceridemia, and it was more prevalent among men (29.4%) than women (20.7%; *p* < 0.001).

### 3.6. Glucose

The mean TC was 5.4 ± 1.1 mmol/L (5.5 ± 1.1 in men vs. 5.3 ± 1.0 mmol/l in women, *p* < 0.001). Fasting hyperglycemia was documented in 26.6% of the investigated population, with a statistically significant difference between sex groups (*p* < 0.001). The glucose level 7.0 mmol/L and above was in 4.5% of all study population with non-significant sex differences (*p* = 0.168). The prevalence of all parameters showed a trend to increase with age in both sex groups (Table 5).

### 3.7. Changes in the Cardiovascular Risk Profile over the Past Decade

**Figure 2:**
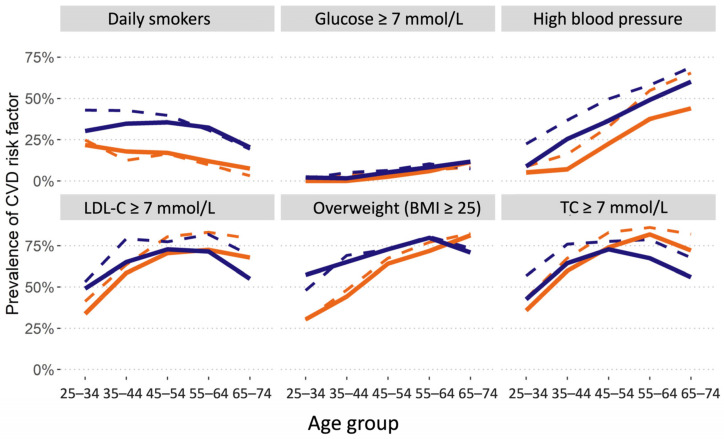
Comparison of the risk factors prevalence in 2 surveys by gender/age. Orange—female, blue—male; Bold—2019, dashed—2009; LDL-C—low-density lipoprotein cholesterol level; BMI—body mass index; TC—total cholesterol level.

The prevalence of the most important RFs was compared with the data from a similar study performed in 2009–2010 [2]. The mean number of five most important cardiovascular RFs has decreased by 0.3 in 10 years, t(5883) = −7.2, *p* < 0.001 in general population, as well as in men and women (Table 6). Table 7 demonstrates the percentage of people without any of the traditional risk factors. There is a positive tendency to increase the percentage of people with zero risk factors in 10 years. Changes in the prevalence of most common RFs were significant (high blood pressure, TC, LDL, HDL, Glu, daily smoking) (Table 8, Figure 2).

The data presented in Figure 2 demonstrate the positive dynamics of the prevalence of several most important RFs over ten years: a decrease in the prevalence of daily smokers, men, in the age group 25–34, from 42.89% in 2009 to 30.25% in 2019; a decrease in the prevalence of TC ≥ 5.0 mmol/L, LDL-C ≥ 3.0 mmol/L and blood pressure in all sex/age groups, and stabilization in the prevalence of Glu ≥ 7.0 mmol/L.

## 4. Discussion

Limited information can be obtained from the morbidity data of the routine outpatient health statistics reports and public health databases to assess disease prevalence. This is due to insufficient information about the causes of diseases and their contributing RFs, and the association between these factors in the general population. Considering this information, epidemiological studies present an opportunity to estimate the prevalence of RFs and thus assist in planning resources and improving prevention strategies. Therefore, conducting a nationwide cross-sectional population survey was of great interest in getting reliable information on the present cardiovascular RFs’ profile of the adult population in Latvia in comparison to the previous survey in 2009–2010.

The major strength of the survey was the large representative sample of the general population from all regions of the country, and the results were representative of the adult population in Latvia. The study sample of 4070 respondents had a slight excess of women (50.5%). The level of distribution in all age groups of both sex groups was optimal or very close to optimal, as shown in Table 1.

The obtained results confirmed the study hypothesis of a high prevalence of cardiovascular risk factors in the Latvian population. A high number of risk factors in the young male population is an unfavorable finding showing the direction for future preventive measures.

Numerous epidemiological studies have identified smoking as one of the most important RFs for non-communicable diseases: cardiovascular, lung diseases, and cancer. The prevalence of smoking in Latvia is rather high. Almost one-third of males and about 15% of females are daily smokers. The finding that smoking is more prevalent in young and middle-aged groups might reflect the contemporary smoking habit in adolescence in general. A proportion of 82.2% of the respondents who had been smokers for at least one year during their lives had started smoking before the age of 21, when buying cigarettes is prohibited by law. The data indicate an urgent need for exact changes in the public health policy, for the target groups and in the tactics of anti-smoking campaigns for teens and grade-schoolers, to prevent the first cigarette at a young age or ever. At the same time, the tobacco policy should include attempts to increase actual smoking cessation among the adult population, as only a half of the smokers report any serious attempts to quit. Overall, 36.8% of all participants were hypertensive. The prevalence among men was 40.3%, and among women 33.8%. The proportion of subjects with hypertension increased considerably with age in both sex groups. The prevalence of hypertension seems to be similar to the data of age-specific prevalence of hypertension in women and men aged 40–79 from national surveys in several European countries [9]: 40% of men and 36% of women in the UK (2016), 46% of men and 43% of women in Germany (2008–2011) are recognized as hypertensive. When comparing the data of elderly respondents (over 60 years of age), the rates of hypertension in Latvia, Finland, Italy, and Ireland are quite similar: 61–70% for women and 64%–72% for men. This underlines once again that the high burden of hypertension is a major health challenge because it increases morbidity and mortality from cardiovascular and kidney diseases, and the financial costs to society [12]. It has been estimated that substantial stroke and ischemic heart disease morbidity and mortality worldwide are attributable to hypertension [12]. Both the primary prevention and the implementation of innovative, cost-effective, and sustainable programs for hypertension prevention and control should be a public health priority in Latvia as the prevalence of some of the contributing factors, such as overweightness and obesity, has remained consistently high over the last two decades [2].

The next strength of this study is the possibility to compare the data with the previous similar study in 2009–2010. Particularly, the high prevalence of obesity and overweightness demonstrated in both studies, as well as the accumulation of other RFs in the middle age groups (35–54 years old), are likely to keep stable the burden of CVD. However, the prevalence of hyperglycemia (fasting blood glucose level ≥ 5.6 mmol/L) has decreased over the last ten years, likely due to the State-supported Patient Educational Programme in Latvia and consistent adherence to the recommendations from the Diabetes Mellitus Treatment Guidelines (2007 and 2016). To some extent, the Health Behaviour among Latvian Adult Population study [13] confirms this.

According to the survey data in 2009, 31.8% of the respondents had their blood glucose tested in the last year, of which 36.5% were women and 25.3% were men. However, a high proportion of adults had never determined their blood glucose level (29.4%). The data from the study in 2019 show significant dynamics: blood glucose levels during the last year were measured in 43.8% (in 51.2% of women and 35.6% of men), and only 12% of the respondents had never been tested.

Attention should be paid to the dyslipidemia profile in this study, especially in comparison to 2009–2010. Although the prevalence of increased levels of TC and LDL-C were comparatively high, the prevalence increased with age in both sex groups. It was interesting to find an improvement of the lipid profile in the age group 65–74, especially in men, when compared to younger age groups. In the previous survey, a similar finding was explained by the death of people with a very high level of RFs in the middle-aged group, especially among men [2]. To note, the male life expectancy at birth in Latvia in 2009 and 2019 was 68.3 years and 70.8 years, respectively. However, a more important finding concerning the lipid profile was the significant decrease in the prevalence of high TC and LDL-C ≥ 3.0 mmol/L over the last ten years: the prevalence of increased TC from 71.8% in 2009 to 63.2% in 2019 (from 71.2 to 60.72% in men and from 72.4 to 65.35% in women); the prevalence of increased LDL-C ≥ 3.0 mmol/L from 70.6% in 2009 to 62.03% in 2019 (from 71.7 to 63.08% in men and from 69.6 to 61.12% in women). This fact is definitely related to the consistent use of statin therapy in CVD patients and the increasing frequency of lipid detection in the population. According to the EUROASPIRE IV survey in 2013–2015, statins were used by 93.1% of the Latvian CVD patients in the set of secondary prevention [14].

According to the data of regular surveys “Health Behaviour among Latvian Adult Population”, in 2008 the blood cholesterol level during the last year was measured by 28% of adults (22.5% men; 31.9% women) while ten years later by 41% of the adult population (34.1% men; 48.5% women) [13]. There has also been a significant reduction in the number of the respondents who were never tested for TC levels: from 41.9% (45.6% men; 39.2% women) in 2008 to 14.2% (17.5% men, 11.1% women) in 2018.

Analyzing the dynamics of AH over ten years (2009–2019), we recognize the statistically significant decrease in the prevalence as being positive. A particular emphasis should be put on the reduction of the AH prevalence in the age groups 25–44 and 35–44. For example, during the study of 2009–2010, the prevalence of AH in men aged 25–34 was 23.9%, and in women 9.2%, in the 2019–2020 study, 10% of men and 5.1% of women were recognized as hypertensive. Similar dynamics were also in the older age groups, i.e., in the age group of 65–74 years old, 77.1% of men and 80% of women were recognized as hypertensive in 2009, and 71.6% of men and 67.4% of women in 2019. However, the analysis of the prevalence of AH in various age groups confirms the fact that the prevalence of arterial hypertension increases with age and, similarly to Latvia, is observed in other European countries. For example, in Finland, 82% of women over the age of 70 are recognized as hypertensive. In Italy, Germany, Ireland, 75–79% of women of the same age group have AH. 69–77% of men over the age of 70 in these countries have AH [15]. However, the high prevalence of AH in this age group is apparently associated with hypertension control problems. Thus, the Latvian health system should set ambitious targets to improve the awareness of hypertension, treatment, and control to prevent the high burden of uncontrolled hypertension.

The dynamics of regular smoking over the last ten years are unsatisfactory, as the prevalence of daily smokers in the women group has even slightly increased. However, it should be noted that the number of young male (25–34 y) smokers has decreased significantly (from 46.3 to 30.3%), while the prevalence rate of female smokers of the same age has not significantly reduced.

Summarizing the results of the nationwide epidemiological studies, it is necessary to stress their importance for the management of public health policy. Like a traffic light, their result package indicates the correct course of action, gives a warning, or raises the alarm.

They point to the tools that need to be used to influence public health and heart health and implement the principles of the European Guidelines on Cardiovascular Disease Prevention [3] and ESC Consensus and Position Papers on Prevention and Rehabilitation in our country. For example, the epidemiological study results of 2009–2010 were used in formulating the Cardiovascular Health Improvement Action Plan for 2013–2015, approved by the Cabinet of Ministers of the Republic of Latvia. Furthermore, the findings of the last survey in 2019–2020 have been incorporated into the document “Public Health Guidelines 2021–2027” in Latvia.

Some limitations should be acknowledged in the study. The level of nonresponse is a common problem in the world of science, and this study was not an exception. A pretty high difference in the response rate was found between those who agreed for interviews and those who agreed also for laboratory tests. The analysis of the prevalence of main risk factors for both groups was performed (all respondents vs. respondents with laboratory tests) and there was no significant difference in the characteristics of the population. BP measurements were taken three times after a rest of at least 5 min. It does not correspond to the hypertension diagnostic algorithm, requiring at least two BP measurements on separate occasions. This fact may cause the overestimated prevalence of hypertension.

## 5. Conclusions

Although the prevalence of cardiovascular RFs remains noteworthy, an improvement in the risk profile of the Latvian population has been observed over the past decade. The study shows subjective self-underestimation of cardiovascular risk. The nationwide comparative data obtained from population-based cross-sectional epidemiological studies of CVD RFs should be used as a baseline against which other measurements can be compared, including monitoring cardiovascular health prevention measures. Based on the prevalence data of a particular RF or a group of RFs in the population, it is possible to model their dynamics and their impact on morbidity and mortality over a period of time as part of specific preventive measures.

## Figures and Tables

**Table 1:** Distribution of the participants by age and sex.

Parameter	Men, *n* (%)	Women, *n* (%)	Total, *n* (%)
**Age group**			
25–29	190 (9.4%)	197 (9.6%)	387 (9.5%)
30–34	195 (9.7%)	220 (10.7%)	415 (10.2%)
35–39	206 (10.2%)	197 (9.6%)	403 (9.9%)
40–44	193 (9.6%)	201 (9.8%)	394 (9.7%)
45–49	197 (9.8%)	204 (9.9%)	401 (9.9%)
50–54	192 (9.5%)	201 (9.8%)	393 (9.7%)
55–59	214 (10.6%)	210 (10.2%)	424 (10.4%)
60–64	214 (10.6%)	210 (10.2%)	424 (10.4%)
65–69	212 (10.5%)	199 (9.7%)	411 (10.1%)
70–74	203 (10.1%)	215 (10.5%)	418 (10.3%)
**Total**	2016	2054	4070

**Table 2:** Socio-demographic characteristics of the s+ tudy population.

Parameter	Men, *n* = 2016	Women, *n* = 2054	Total, *n* = 4070
**Age group**			
**Education, *n* (%)**			
Higher (1st/2nd level)	495 (24.6%)	818 (39.8%)	1313 (32.3%)
Primary	271 (13.4%)	202 (9.8%)	473 (11.6%)
Secondary	945 (46.9%)	715 (34.8%)	1660 (40.8%)
Secondary professional	305 (15.1%)	319 (15.5%)	624 (15.3%)
**Marital status, *n* (%)**			
Divorced	221 (11.0%)	264 (12.9%)	485 (11.9%)
Married	1349 (66.9%)	1243 (60.5%)	2592 (63.7%)
Never- married	399 (19.8%)	321 (15.6%)	720 (17.7%)
Widowers/widows	47 (2.3%)	226 (11.0%)	273 (6.7%)
**Occupation status, *n* (%)**			
Housewife/Househusband	26 (1.3%)	173 (8.4%)	199 (4.9%)
Jobless	129 (6.4%)	94 (4.6%)	223 (5.5%)
Laborers	934 (46.3%)	522 (25.4%)	1456 (35.8%)
Retired	430 (21.3%)	439 (21.4%)	869 (21.4%)
Students	4 (0.2%)	10 (0.5%)	14 (0.3%)
White-collar workers	493 (24.5%)	816 (39.7%)	1309 (32.2%)

**Table 3:** Self-reported risk factors and medical treatment of the study population.

Parameter	Male, *n* = 2016	Female, *n* = 2054	Total, *n* = 4070	*p*-Value, Men vs. Women
Risk factors, *n* (%)				
Hypertension	15.9 (14.4, 17.6)	20.9 (19.2, 22.8)	18.6 (17.4, 19.9)	<0.001
High cholesterol	15.4 (13.8, 17.1)	22.7 (20.8, 24.7)	19.3 (18.0, 20.6)	<0.001
Diabetes	4.5 (3.6, 5.4)	6.1 (5.1, 7.3)	5.3 (4.6, 6.1)	0.023
Current smoking	40.4 (38.1, 42.7)	21.0 (19.2, 22.9)	30.0 (28.5, 31.5)	<0.001
Physical inactivity	8.6 (7.4, 9.9)	12.0 (10.7, 13.5)	10.4 (9.5, 11.4)	<0.001
Medical treatment, *n* (%)				
Anti-hypertensive agents	15.5 (14.0, 17.2)	20.5 (18.7, 22.3)	18.2 (17.0, 19.4)	<0.001
Anti-cholesterol agents	8.0 (6.9, 9.2)	12.5 (11.1, 14.1)	10.4 (9.5, 11.4)	<0.001

**Table 4:** Prevalence of the observed CVD risk factors in 2019–2020.

Parameters	All Population *n* = 2218		Men *n* = 976		Women *n* = 1242		Men vs. Women
Risk factor	%	[95% CI]	%	[95% CI]	%	[95% CI]	*p*
TC, ≥5 mmol/L	63.2	[61.0, 65.3]	60.7	[57.3, 64.0]	65.4	[62.5, 68.1]	0.036
LDL-C, <2 mmol/L	7.9	[6.8, 9.2]	7.8	[6.2, 9.8]	8.0	[6.5, 9.8]	0.870
LDL-C, 2–2.99 mmol/L	30.1	[28.0, 32.2]	29.1	[26.1, 32.3]	30.9	[28.2, 33.7]	0.406
LDL-C, ≥3 mmol/L	62.0	[59.8, 64.2]	63.1	[59.7, 66.3]	61.1	[58.2, 63.9]	0.379
HDL-C, ≤ 1 (1.2) mmol/L for men (women)	14.0	[12.5, 15.6]	16.9	[14.5, 19.5]	11.5	[9.8, 13.5]	0.001
Tg, ≥1.7 mmol/L	24.7	[22.9, 26.7]	29.4	[26.4, 32.5]	20.7	[18.5, 23.2]	<0.001
Glu, 5.6–6.99 mmol/L	22.1	[20.4, 24]	27.1	[24.2, 30.1]	17.8	[15.7, 20.1]	<0.001
Glu, ≥7 mmol/L	4.5	[3.7, 5.5]	5.2	[3.9, 6.7]	4.0	[3.0, 5.2]	0.168
High blood pressure (≥140 and/or 90)	28.0	[26.1, 30.0]	33.3	[30.2, 36.4]	23.5	[21.1, 26.0]	<0.001
Arterial hypertension	36.8	[34.7, 38.9]	40.3	[37.0, 43.6]	33.8	[31.2, 36.6]	0.003
Overweight (BMI 25–29.9)	34.3	[32.2, 36.4]	40.6	[37.3, 44.0]	28.8	[26.3, 31.5]	<0.001
Obesity (BMI ≥ 30)	29.1	[27.1, 31.1]	28.2	[25.3, 31.3]	29.8	[27.3, 32.5]	0.427
Have smoked at least for 1 year during lifetime	45.3	[43.1, 47.5]	60.8	[57.5, 64.1]	31.7	[29.1, 34.5]	<0.001
Daily smokers	22.8	[21.0, 24.7]	31.5	[28.5, 34.8]	15.2	[13.2, 17.4]	<0.001

TC—total cholesterol; LDL-C—low-density lipoprotein cholesterol; HDL-C—high-density lipoprotein cholesterol; Tg—triglycerides; Glu—fasting glucose; BMI—body mass index.

**Table 5:** Prevalence of the observed CVD risk factors in 2019–2020 by sex/age (%).

Age Groups	25–34		35–44		45–54		55–64		65–74	
Sex	M	W	M	W	M	W	M	W	M	W
TC, ≥5 mmol/L	42.5	35.7	64.5	59.8	72.9	74.0	67.5	81.9 ***	56.0	72.2 ***
LDL-C, <2 mmol/L	10.9	14.8	3.2	5.9	6.5	3.8	6.9	5.5	13.8	10.8
LDL-C, 2–2.99 mmol/L	40.0	51.4 *	31.7	35.6	20.7	25.6	21.6	21.9	31.3	21.4 *
LDL-C, ≥3 mmol/L	49.0	33.7 **	65.1	58.5	72.8	70.6	71.5	72.6	54.9	67.8 **
HDL-C, ≤1 (1.2) mmol/L for men (women)	14.6	8.6	13.1	12.2	18.7	7.2 ***	20.8	16.0	17.8	13.3
Tg, ≥1.7 mmol/L	21.2	6.9 ***	26.4	11.6 ***	39.2	18.5 ***	35.9	32.0	22.2	33.4 **
Glu, 5.6–6.99 mmol/L	11.3	5.8	24.6	9.8 ***	31.2	16.2 ***	36.7	27.0 *	37.2	29.1
Glu, ≥7 mmol/L	2.1	0.0	1.6	0.0	5.2	2.6	8.2	6.0	11.7	11.3
High blood pressure (≥140 and/or 90)	8.8	5.1	25.4	7.0 ***	36.5	22.6 **	49.1	37.5 *	60.2	44.0 **
Arterial hypertension	10.0	5.1	28.0	9.4 ***	43.5	32.8 *	64.4	52.9 *	71.7	67.4
Overweight (BMI 25–29.9)	39.9	16.2 ***	40.7	28.0	39.6	32.8	42.2	30.1 **	40.7	37.0
Obesity (BMI ≥ 30)	17.5	14.2	24.4	16.3 *	33.2	31.3	37.6	41.7	30.2	44.3 **
Have smoked at least for 1 year during lifetime	52.7	40.0 *	59.5	36.2 ***	65.2	32.8 ***	67.7	27.3 ***	59.3	22.7 ***
Daily smokers	30.3	21.8	34.8	17.9 ***	35.5	17.0 ***	32.4	11.9 ***	20.3	7.4 ***

M—men; W—women; TC—total cholesterol; LDL-C—low-density lipoprotein cholesterol; HDL-C—high-density lipoprotein cholesterol; Tg—triglycerides; Glu—fasting glucose; BMI—body mass index.* *p* < 0.05, ** *p* < 0.01, *** *p* < 0.001 comparing men vs. women.

**Table 6:** Comparison of the mean number of risk factors between 2009–2010 and 2019–2020 studies.

	2009–2010		2019–2020		2009–2010 vs. 2019–2020		
	Mean	95% CI	Mean	95% CI	*t*	*df*	*p*
All	2.3	[2.3, 2.4]	2.0	[2.0, 2.1]	−7.2	5883	<0.001
Men	2.6	[2.5, 2.7]	2.3	[2.2, 2.4]	−4.5	2298	<0.001
Women	2.1	[2.0, 2.1]	1.8	[1.7, 19]	−6.1	3583	<0.001

Risk factors included: High blood pressure, LDL-C, Glu, BMI, daily smokers.

**Table 7:** Comparison of the population with 0 risk factors between 2009–2010 and 2019–2020 studies.

Age Group	2009–2010		2019–2020	
	Men	Women	Men	Women
**25–34**	15.1%	28.1%	16.2%	35.7%
**35–44**	3.0%	15.4%	4.8%	20.7%
**45–54**	1.1%	4.7%	3.0%	10.4%
**55–64**	1.4%	2.4%	1.4%	3.1%
**65–74**	1.5%	0.8%	2.6%	2.9%
**Total**	5.0%	10.2%	6.1%	14.3%

**Table 8:** Prevalence of the observed CVD risk factors in 2009–2010 and comparison to 2019–2020 (Δ10y).

	All Population			Men			Women		
Risk Factor	%	[95% CI]	Δ10y	%	[95% CI]	Δ10y	%	[95% CI]	Δ10y
**TC, ≥5 mmol/L**	71.8	[70.0, 73.7]	−8.6 ***	71.1	[67.9, 74.2]	−10.4 ***	72.4	[70.2, 74.5]	−7.0 ***
**LDL-C, <2 mmol/L**	4.6	[3.9, 5.6]	+3.3 ***	4.1	[3.0, 5.6]	+3.7 **	5.1	[4.0, 6.4]	+2.9 **
**LDL-C, 2–2.99 mmol/L**	24.8	[23.0, 26.6]	+5.3 ***	24.2	[21.2, 27.4]	+4.9 *	25.3	[23.3, 27.4]	+5.6 **
**LDL-C, ≥3 mmol/L**	70.6	[68.6, 72.4]	−8.6 ***	71.7	[68.4, 74.8]	−8.6 ***	69.6	[67.4, 71.8]	−8.5 ***
**HDL-C, ≤ 1 (1.2) mmol/L for men (women)**	17.0	[15.5, 18.6]	−3.0 **	19.6	[17.0, 22.4]	−2.7	14.9	[13.3, 16.5]	−3.4 **
**Tg, ≥1.7 mmol/L**	26.9	[25.2, 28.7]	−2.2	34.0	[30.9, 37.2]	−4.6 *	21.0	[19.2, 22.8]	−0.3
**Glu, 5.6–6.99 mmol/L**	26.8	[25.2, 28.5]	−4.7 ***	30.8	[27.9, 33.8]	−3.7	23.4	[21.6, 25.3]	−5.6 ***
**Glu, ≥7 mmol/L**	5.0	[4.2, 5.8]	−0.5	5.7	[4.4, 7.4]	−0.5	4.3	[3.6, 5.2]	−0.3
**High blood pressure (≥140 and/or 90)**	39.3	[37.4, 41.2]	−11.3 ***	44.1	[40.8, 47.4]	−10.8 ***	35.2	[33.2, 37.4]	−11.7 ***
**Arterial hypertension**	45.5	[43.5, 47.4]	−8.7 ***	49.8	[46.3, 53.2]	−9.5 ***	41.9	[39.7, 44.1]	−8.1 ***
**Overweight (BMI 25–29.9)**	37.1	[35.2, 39.1]	−2.8	43.8	[40.5, 47.1]	−3.2	31.5	[29.5, 33.6]	−2.7
**Obesity (BMI ≥ 30)**	26.9	[25.2, 28.5]	+2.2	23.6	[21.0, 26.4]	+4.6 *	29.6	[27.6, 31.6]	+0.2
**Have smoked at least for 1 year during lifetime**	39.2	[37.2, 41.2]	+6.1 ***	58.5	[55.1, 61.7]	+2.3	23.0	[21.0, 25.0]	+8.7 ***
**Daily smokers**	24.2	[22.4, 26.1]	−1.4	37.0	[33.7, 40.3]	−5.5 *	13.4	[11.8, 15.2]	1.8

TC—total cholesterol; LDL-C—low-density lipoprotein cholesterol; HDL-C—high-density lipoprotein cholesterol; Tg—triglycerides; Glu—fasting glucose; BMI—body mass index. Δ10y: change in the prevalence compared to the 2019–2020 study; (+) increase/(−) decrease.* *p* < 0.05, ** *p* < 0.01, *** *p* < 0.001 comparing years 2019–2020 vs. 2009–2010.

## Data Availability

Not applicable.
